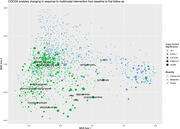# Specific plasma proteins and metabolites convey the effects of lifestyle interventions that improve cognition and function in Alzheimer’s disease

**DOI:** 10.1002/alz.091942

**Published:** 2025-01-09

**Authors:** Jared C. Roach, Cory C Funk, Gwênlyn Glusman, David A. Merrill, John F. Hodes, Molly K. Rapozo, Lance Edens, Ananya Vaidyaraman, William R Shankle, Leroy Hood, Jennifer E. Bramen, the COCOA Consortium

**Affiliations:** ^1^ Institute for Systems Biology, Seattle, WA USA; ^2^ David Geffen School of Medicine at University of California Los Angeles, Los Angeles, CA USA; ^3^ Pacific Brain Health Center, Pacific Neuroscience Institute and Foundation, Santa Monica, CA USA; ^4^ Pacific Brain Health Center, Pacific Neuroscience Institute Foundation, Santa Monica, CA USA; ^5^ Hoag Memorial Hospital Presbyterian, Newport Beach, CA USA; ^6^ Hoag Neurosciences Institute, Newport Beach, CA USA; ^7^ University of California at Irvine, Irvine, CA USA; ^8^ Embic Corporation, Newport Beach, CA USA; ^9^ Shankle Clinic, Newport Beach, CA USA; ^10^ University of California Los Angeles, Los Angeles, CA USA; ^11^ Saint John’s Cancer Institute at Providence Saint John’s Health Center, Santa Monica, CA USA

## Abstract

**Background:**

The Coaching for Cognition in Alzheimer’s (COCOA) Trial was a prospective RCT testing a remotely coached multimodal lifestyle intervention for participants early on the Alzheimer’s disease spectrum. Intervention focused on diet, exercise, cognitive training, sleep, stress, and social engagement. Enrollment criteria targeted individuals with cognitive decline who were able to engage remotely with a professional coach. COCOA demonstrated cognitive and functional benefits. Dense omics data were collected on 53 individuals (≥ 58 years).

**Methods:**

We sought to identify blood analytes that mediated the effects of specific elements of the multimodal intervention on specific outcomes. Outcomes were assessed with the MCI Screen (MCIS) and the Functional Assessment Staging Tool (FAST). We combined these and other measures with proteomics and metabolomics data. We analyzed the resulting dataset of over 300,000 distinct molecular data points—reflecting over 1400 measures— assayed over a period of two years. We used MEGENA to hierarchically multiscale cluster analytes based on correlated responses and identified individual metabolites and functional clusters associated with each intervention and outcome. We analyzed individual time courses of key analyte mediators to illustrate personalized effects of interventions and individualized functional and cognitive outcomes.

**Results:**

Distinct sets of correlated serum analytes (“communities”) convey effects to functional (FAST) outcome and to cognitive (MCIS) outcome. Distinct communities respond to different modalities of intervention. Participants followed different aspects of the multimodal recommendations to different extents, and the analytes in their blood also responded idiosyncratically; analyte trajectories in different individuals show distinct dynamics. We made personalized predictions of future inflections in outcome based on observed changes in key serum mediators. We validated results with data from the Precision Recommendations for Environmental Variables, Exercise, Nutrition and Training Interventions to Optimize Neurocognition (PREVENTION) Trial.

**Conclusions:**

Lifestyle interventions have profound effects on blood metabolites (**Figure 1**). These in turn convey subtler specific effects to cognition and broad‐based effects to function. Pathways that ameliorate the impact of AD via lifestyle interventions in some individuals include nitrogen subsystems, kidney function, and mitochondrial metabolism. These highlight the importance of clinical attention to overall health spanning multiple organ systems in individuals across the Alzheimer’s disease spectrum.